# In developing mouse kidneys, orientation of loop of Henle growth is adaptive and guided by long‐range cues from medullary collecting ducts

**DOI:** 10.1111/joa.13012

**Published:** 2019-05-17

**Authors:** C‐Hong Chang, Jamie A. Davies

**Affiliations:** ^1^ Deanery of Biomedical Science University of Edinburgh Edinburgh UK; ^2^ Yale University School of Medicine, Medicine New Haven CT USA

**Keywords:** chemotaxis, guidance, kidney development, nephrogenesis, tubulogenesis

## Abstract

The path taken by the loop of Henle, from renal cortex to medulla and back, is critical to the ability of the kidney to concentrate urine and recover water. Unlike most developing tubules, which navigate as blind‐ended cylinders, the loop of Henle extends as a sharply bent loop, the apex of which leads the double tubes behind it in a ‘V’ shape. Here, we show that, in normal kidney development, loops of Henle extend towards the centroid of the kidney with an accuracy that increases the longer they extend. Using cultured kidney rudiments, and manipulations that rotate or remove portions of the organ, we show that loop orientation depends on long‐range cues from the medulla rather than either the orientation of the parent nephron or local cues in the cortex. The loops appear to be attracted to the most mature branch point of the collecting duct system but, if this is removed, they will head towards the most mature collecting duct branch available to them. Our results demonstrate the adaptive nature of guidance of this unusual example of a growing epithelium, and set the stage for later work devoted to understanding the molecules and mechanisms that underlie it.

## Introduction

**Figure 1:**
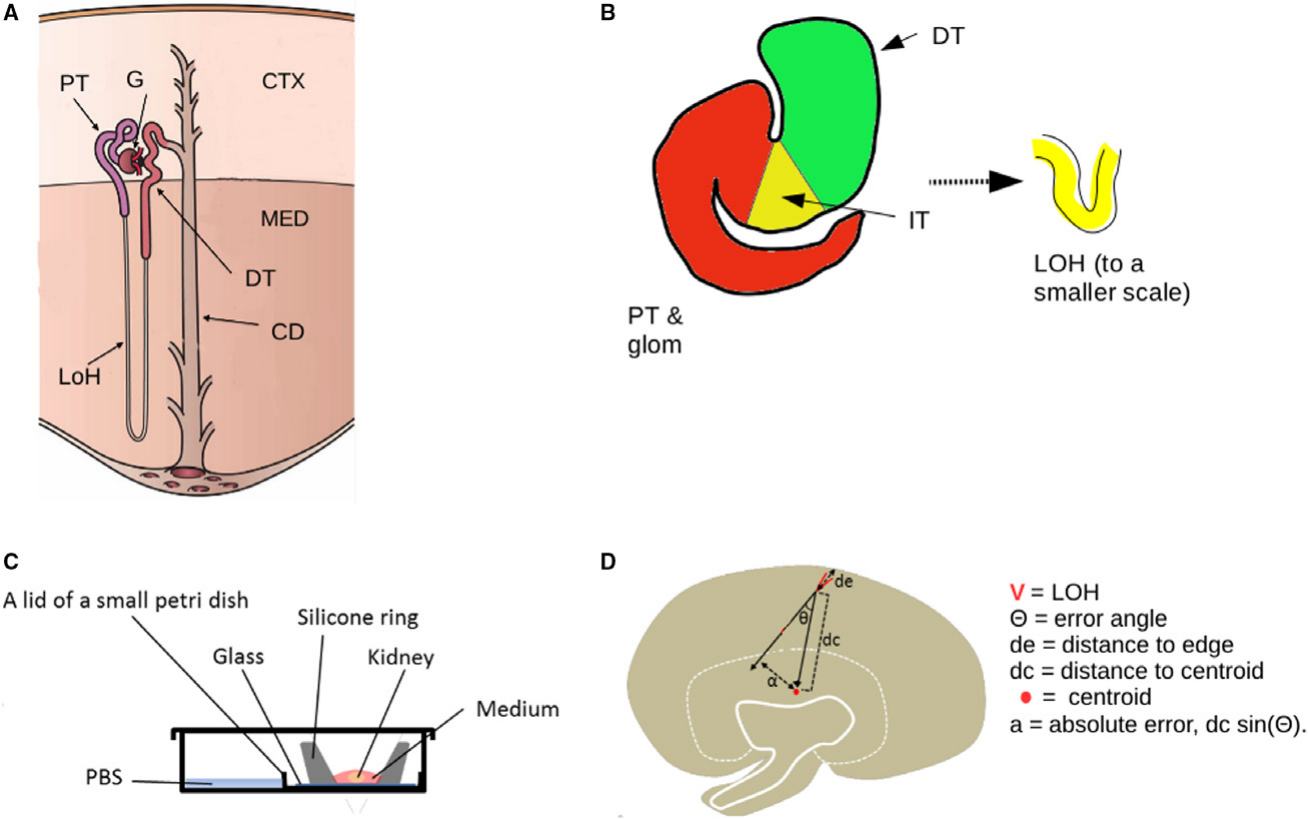
Introduction to nephron anatomy, and illustrations of methods used in this analysis. (A) Route taken by a typical nephron in the kidney. CTX, cortex. MED, medulla; G, glomerulus; PT, proximal tubule; LoH, loop of Henle; DT, distal tubule; CD, collecting duct. (B) S‐shaped tubule stage of nephron development (the name referred originally to the German Gothic character S, later transliterated into the letter ‘S’ to which the tubule bears less resemblance), and the loop emerging from it as a bent tubule. GP, presumptive glomerular podocytes; PT, presumptive proximal tubule; IT, intermediate tubule (presumptive loop of Henle); DT, presumptive distal tubule. This structure places the presumptive distal tubule close to the entrance of the future glomerulus (the slit in the S), where it will remain. The IT will extend away as a loop to form the loop of Henle. (C) Culture system we used and (D) directional measurements made and the ‘error angle’ and ‘absolute error’ calculated from them. Image credit (A): Holly Fischer, Wikimedia Commons, used under the Creative Commons Attribution 3.0, with labels abbreviated.

The motto under the coat of arms of the Anatomical Society, to which this journal belongs, is *ex conformatione usus*: from structure comes function. The link between structure and function is particularly clear in the metanephric kidney, in which the ability of the body to recover water depends critically on the gross anatomy of the organ. Specifically, it depends on the division into cortex and medulla, and on the paths taken by fine tubules as they cross the boundaries between these gross divisions (Fig. 1A). There are tens of thousands of these tubules (nephrons) in mouse kidneys and around a million in human kidneys.

The glomeruli of the kidney are located in its cortex, where they filter blood to produce a primary filtrate approximately isotonic with extracellular fluid. This filtrate passes through the proximal convoluted tubule, where approximately 65% of salts and other solutes are recovered, and a corresponding proportion of water follows the salts by a transcellular route, being drawn by the osmotic effect of the salt recovery. The filtrate, still isotonic with body fluids, then enters the loop of Henle, which dips into the strongly hypertonic renal medulla. The descending limb of the loop of Henle is permeable to water, which is drawn into the hypertonic interstitium, leaving the filtrate in the tube more concentrated. In the ascending limb, salt is actively recovered from the filtrate and passed into the interstitium, maintaining its hypertonic nature. The loops of Henle of other nephrons enter this same medulla, so they all contribute collectively to its hypertonicity. So efficient is the salt recovery process that the urine that leaves the loop and re‐enters the cortex is hypotonic compared with even cortical interstitium, and more water is recovered osmotically as it passes along the distal tubule. The distal tubule passes very close to the glomerulus that feeds it (Fig. 1A). Specialized structures (macula densa and juxtaglomerular apparatus) where the distal tubule contacts the glomerulus, enable physiological measurements of the osmolarity of the urine in the distal tubule. These measurements generate error‐correcting signals that control local blood flow into the glomerulus, a process called tubuloglomerular feedback. The urine then enters the collecting duct system, which passes once more through the hypertonic medulla, where either some or most of the remaining 25% of the originally filtered water is recovered into the hypertonic interstitium through physiologically regulated aquaporin channels in collecting duct cells (see Danziger et al. 2012, for a review of this renal physiology).

It follows from the above description that the ability of kidneys to recover water depends on the loops of Henle extending accurately into the medulla, so that their salt recovery makes the medulla, but not the cortex, hypertonic. The need for growing tubules to navigate accurately in development is common; navigation by developing tracheae of *Drosophila melanogaster* and developing branches of blood vessels have been particularly well studied (Maruyama & Andrew, 2012; Gerhardt & Betsholtz, 2005). Generally, these tubules grow as simple projections, the blind end of a tube advancing into surrounding tissues and the tubular shaft following behind. The development of the loop of Henle, however, is subject to the severe constraint that its return path from the medulla has to be toward the glomerulus of its own nephron in order that the distal tubule can perform local tubuloglomerular feedback. A blind‐ended tube navigating down to the medulla and then returning to find its own glomerulus, among thousands of others nearby, is probably not feasible. What actually happens is that the presumptive glomerulus, proximal tubule, intermediate tubule (presumptive loop of Henle) and distal tubule develop as a compact epithelial structure, the ‘S‐shaped tubule’ (Fig. 1B), which brings the presumptive distal tubule very close to the glomerulus. The loop of Henle emerges as a bending‐out from this S‐shaped tubule, and grows as an elongating loop, rather like the extending slide of a trombone. This looping growth does not disturb the relationship between distal tubule and glomerulus, so the problem of navigating to the correct glomerulus never arises (see Saxén, 1987 for a general review of the anatomy of renal development).

Navigation by a loop is unusual in development, and little is known about how it works. In this paper, we report basic experiments on loop of Henle navigation. The results suggest that direction is set neither by the orientation of the S‐shaped tubule that gives rise to the loop nor by factors in the local cortex. Rather, it is orientated by long‐range signals from the medulla, specifically from the deepest and most mature collecting ducts or their associated stroma.

## Materials and methods

### Embryonic mouse kidneys

Pregnant CD1 mice were raised, mated and sacrificed according to a method listed in Schedule 1 of the Animal Scientific Procedures Act (1986) by a Home Office licensed facility at the University of Edinburgh. Staging of pregnancy was calculated on the assumption that the morning of discovery of a vaginal plug = E0.5, and was checked for each embryo using limb morphology as a criterion. Kidney rudiments from E11.5 embryos were harvested for culture as described in Davies (2010). Kidney rudiments from E14.5–E16.5 embryos were isolated by micro‐dissection using 25‐gauge needles.

### Cryosectioning

Embryonic kidneys were fixed in 4% formaldehyde (made freshly from paraformaldehyde) in phosphate‐buffered saline (PBS) overnight at 4 °C, rinsed several times in PBS, incubated in 30% sucrose in PBS overnight at 4 °C and then in a 1 : 1 mix of 30% sucrose in PBS/optimal cutting temperature (OCT) embedding matrix (Thermo Scientific 12678646) for a further 24 h. They were embedded in the same mixture and frozen using dry ice. Sections were cut at 12 μm on a Leica CM3050S cryostat, transferred to polylysine‐coated slides (Fisher 1255015), air‐dried for an hour, and either used directly or stored with silica gel desiccant at −20 °C.

### Kidney organ culture

Organ culture was performed using a minor modification of the Sebinger culture system (Sebinger et al. 2010), the culture volume being 82 μL for the first 2 days and 85 μL thereafter. Culture medium was Eagle's minimal essential medium (MEM) with Earle's salts (Sigma M5650), supplemented with 10% foetal bovine serum (Invitrogen 10108165) and 1% penicillin/ streptomycin stock solution (Sigma P4333). It was changed every 2 days. The culture system is depicted in Fig. 1C. Cutting and removing or rotating cut pieces was performed under a dissecting microscope using 25‐gauge hypodermic needles as dissecting instruments.

### Immunofluorescence

Cultured organs were fixed by removal of their medium and immersion in methanol pre‐cooled to −20 °C. They were allowed to warm to room temperature over 10–15 min, and the methanol was replaced by PBS for 30 min at room temperature. Primary antibodies were diluted in PBS as follows: rabbit anti‐laminin, Sigma L9393, 1 : 100; chicken anti‐laminin, Abcam ab14055, 1 : 500; mouse anti‐calbindin, Abcam ab9481, 1 : 200; rabbit anti‐THP, Bioquote bt‐590, 1 : 100. They were applied overnight at 4 °C, then rinsed off with PBS for several hours before secondary antibodies were applied. These were also dissolved in PBS at 1 : 100 and were as follows: goat anti‐chicken FITC, Abcam ab97134; goat anti‐chicken TRITC, Abcam ab6874; goat anti‐mouse FITC, Sigma F2012; goat anti‐rabbit TRITC, Sigma T6778; sheep anti‐rabbit FITC, Chemicon AB7130F; horse anti‐mouse AMCA, Vector CI‐2000. Secondary antibodies were applied overnight at 4 °C, and samples were washed for a few hours at room temperature the following day before being mounted in 50% PBS/50% glycerol. 22 × 22 mm coverslips were used as pillars to support an overall 22 × 64 mm coverslip over the samples to prevent the samples being crushed. Imaging was performed on a Zeiss epifluorescence microscope.

Cryosectioned embryonic material was permeabilized in 0.1% Triton X‐100 in PBS (TPBS) for 20 min, then stained in primary antibodies (diluted as above, but in TPBS) overnight at 4 °C. The sections were rinsed in TPBS for 30 min at room temperature, then secondary antibodies diluted in TPBS were applied. The following day, samples were washed in TPBS for 30 min and examined using a Zeiss epifluorescence microscope.

### Measurement of loop of Henle orientation

Loop of Henle orientation was measured with respect to the centroid of the kidney (the point on which a two‐dimensional section of it could be balanced): this calculable point of reference was used to avoid subjective judgements about where the ‘centre’ of each kidney was. To locate the centroid, images of kidney sections, or of whole‐mounts of cultured kidneys, were imported into imagej, their outline was drawn round manually, and the imagej ‘centroid’ function was used to calculate the point. For each loop of Henle, a straight line (the ‘current course’ line) was drawn from its apex to the mid‐point between its two limbs (Fig. 1D) and extended forward beyond the apex. The ‘error angle’, Θ, was calculated as the angle between a line drawn from the centroid to the loop of Henle's apex, and the ‘current course’ line (Fig. 1D). For some samples, the closest approach between the ‘current course’ line and the centroid was measured as the ‘absolute error’, α. This α is related to Θ and the distance, dc, between the apex and the centroid, by the formula α = dc sin Θ (Fig. 1D).

## Results

### Loops of Henle orientate towards the renal centre in foetal mouse kidneys

The loops of Henle of an adult metanephric kidney are orientated radially, to run from the cortex into the medulla and then return (see Introduction). Our first question was whether this orientation is established as soon as the loops are detectable in foetal development or is established by later remodelling. This possibility, of initial development in one pattern followed by later remodelling to create another, cannot be dismissed without checking: the collecting duct tree, for example, develops first in a fractal manner and achieves its later, largely radial form in the medulla through remodelling (branch node retraction: Lindström et al. 2015). The presence of loops of Henle early in kidney development has already been noted (e.g. Georgas et al. 2011); we set out to address the specific question about accuracy of orientation towards the medulla.

**Figure 2:**
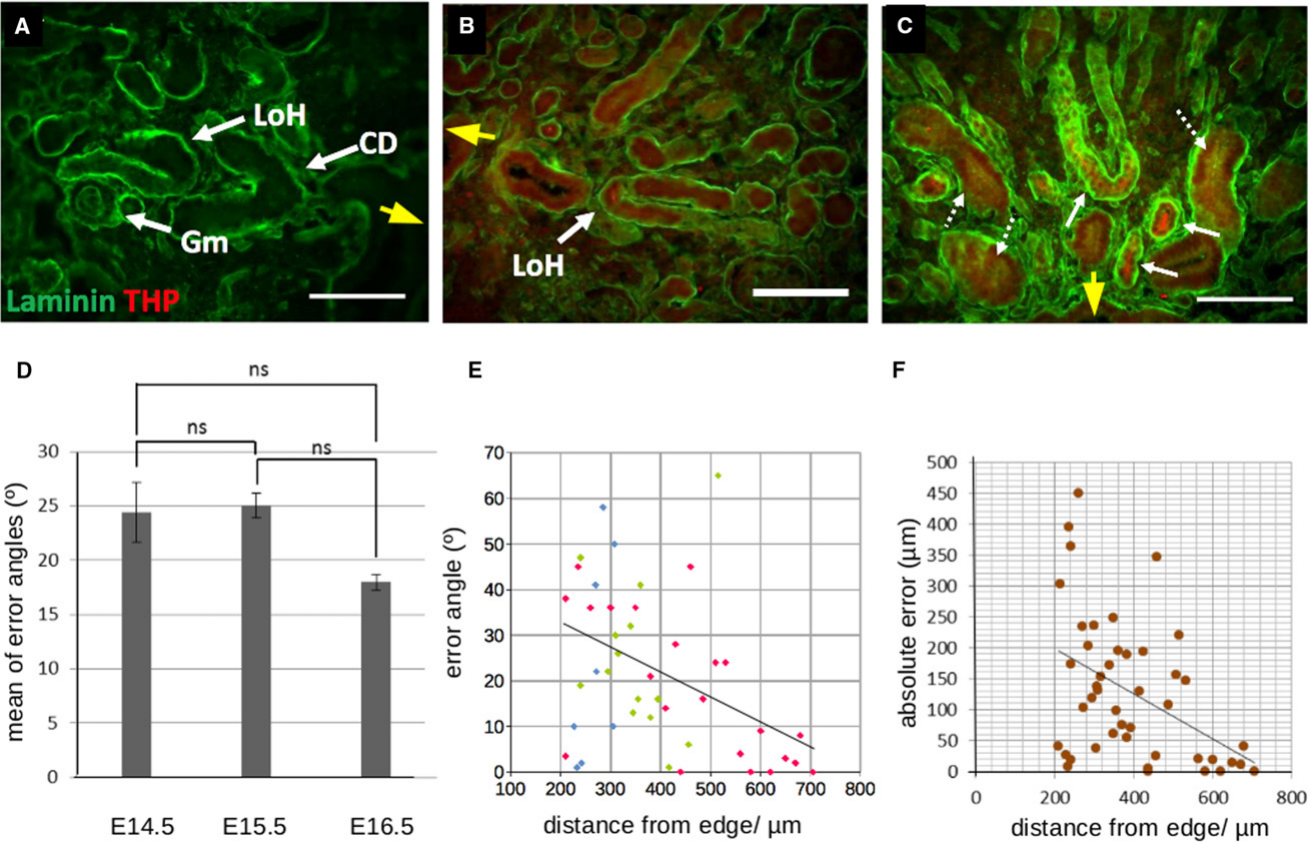
Loop of Henle development *in vivo*. Images (A,B,C) show loops of Henle in E14.5, E15.5 and E16.5 kidneys, respectively, stained for laminin and THP. The approximate direction from the centre of the image toward the centre of the kidney is shown by a yellow arrow, pointing toward the centre of the kidney. In E14.5 kidneys, short loops of Henle (LoH) are present, together with glomeruli (Gm), collecting ducts (CD) and other parts of the nephron. At this age, none express THP. By E15.5, the loops express THP, as they do at E16.5 (solid arrows), and weak THP staining is also seen in collecting ducts (dotted arrows). (D) Mean error angle in loops of Henle at different ages (ns = difference cannot be proved at *P* = 0.05). (E) Plot of the error angle of loops against the distance of their tip from the edge of the kidney, a proxy for their maturity as explained in the text. Blue points are from E14.5 kidney, green from E15.5 and red from E16.5. The trend line is from all points considered together. (F) Plot of the absolute error – the distance by which a loop would miss the centroid of the kidney if it maintained its current course – against the distance of the tip from the edge of the kidney. Both measures show that orientation becomes more accurate as more mature nephrons are examined. Scale bars: 100 μm.

To visualize loops of Henle in naturally developing kidneys, we isolated kidney rudiments from E14.5, E15.5 and E16.5 mouse embryos, cryosectioned them, and stained them with an antibody against laminin to mark basement membranes of all tubules, and an antibody against Tamm‐Horsfall Protein (THP) to mark the maturing loop of Henle (Tamm & Horsfall, 1950; Pennica et al. 1987). It is, however, known that THP is absent at the very earliest stage of loop formation, when morphology alone has to be used for identification (Nakai et al. 2003). At E14.5, five of a total of eight kidneys examined exhibited very short *U*‐shaped loops of Henle but they expressed no THP (Fig. 2A). In the other three of the eight kidneys, no loops could be discerned. E15.5 kidneys exhibited longer and straighter loops of Henle (in nine of nine kidneys examined), and THP was expressed by the loop (Fig. 2B). At E16.5, nine of nine kidneys examined exhibited clear THP‐positive loops of Henle. Some of these loops reached the innermost parts of the medulla (Fig. 2C).

To examine orientation of loops quantitatively, we defined the centroid of the kidney as a reference point (see Materials and Methods) and measured the ‘error angle’ by which the orientation of each loop of Henle diverged from following a radial line directly to the centroid. An error angle of 0° would mean a perfectly centripetal alignment, 90° would mean circumferential rather than radial growth, and 180° would mean centrifugal growth. Our measurements did not discriminate between errors to the left or right of the radial line (so two loops, one with an error 45° to the left and the other with an error 45° to the right, would yield a mean error of 45°, not 0°). The loops of E14.5 kidneys (*n* = 8) showed a mean error angle of 24.4 ± 2.7°, those of E15.5 kidneys (*n* = 15) 25 ± 1.1° and those of E16.5 (*n* = 22) kidneys 18 ± 0.7° (Fig. 2D: in all these cases ‘±’ terms denote standard error of the mean). There was no apparent difference between accuracy of the two younger stages. The E16.5 samples showed a somewhat lower mean error angle but a null hypothesis that there was no difference between this and the earlier kidneys could not be rejected at *P* < 0.05. Pooled data from all ages showed that 85% of these loops of Henle showed an error of less than 45°, providing clear evidence of non‐random orientation.

During renal development, new nephrons form near the ureteric bud tips in the cortex as the cortex advances with organ growth (Herring, 1898). Foetal kidneys therefore show a spatial gradient of nephron maturity, the most mature being deeper and the least mature being superficial. We exploited this relationship between location and maturity to test whether loops of Henle orientate correctly at the start of their development or achieve their orientation only later, by plotting the navigation error of a loop of Henle against the distance of that loop from the edge of the kidney, as a proxy for its age. A simple plot of the error angle, as previously defined, shows a trend of a gently decreasing error with distance (Fig. 2E). It is noticeable that the youngest loops (closest to the outer edge of the kidney) are comparably inaccurate at all ages, and accuracy becomes generally better, with occasional outliers, with loop age/distance in both E15.5 and E16.5 kidneys; E14.5 kidneys are too small to show this effect; their data points occupy only the left end of the *x*‐axis. There is, however, a problem in interpreting data expressed in this manner. Seen from the point of view of the tip of loop of Henle itself, the navigational significance of an error of given angular size is greater, the further away from its goal that tip is: the further away something is from its target when it makes an uncorrected error, the greater the absolute distance by which it will miss that target. We therefore calculated the ‘absolute error’ of each tip – that is, the distance by which it would miss the renal centroid if it carried on along its current course uncorrected. This absolute error also showed a trend for the more central, older loops to be the most accurate (Fig. 2F). Loops of Henle developing *in vivo* therefore show robust centripetal orientation, which becomes more accurate as the loops elongate and mature.

### Similar loop of Henle orientation can be seen in organ culture

**Figure 3:**
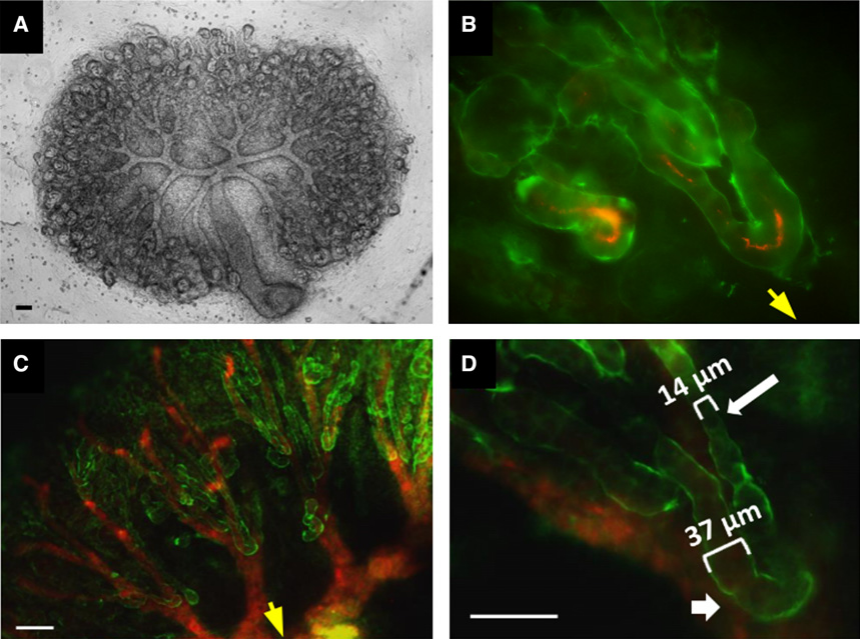
Development of kidney rudiments in organ culture. (A) Bright‐field image of a cultured organ. (B) Loop of Henle in such a culture, the laminin‐rich basement membrane shown in green and THP in red. (C) View of multiple loops of Henle growing approximately centripetally in a culture kidney: it is noticeable that the loops (green, due to anti‐laminin stain) tend to associate with (be found running on or alongside) the collecting ducts (red, due to anti‐calbindinD^28k^ stain). (D) A high‐power view of a loop of Henle in organ culture shows distinct thick and thin segments (indicated with a short, thick arrow and a long, thin arrow, respectively): as in (C), the red stain is anti‐calbindinD^28k^, not THP. Scale bars: 100 μm (A,C,D), 50 μm (B). Yellow arrows as in Fig 2.

We have previously described a culture system, ‘Sebinger culture’, that allows kidney rudiments and organoids to grow large, flat and to an advanced stage of maturity that includes the presence of loops of Henle (Sebinger et al. 2010; Chang & Davies, 2012; Elhendawi & Davies, 2018). We again verified that E11.5 kidneys, isolated from mouse embryos and grown for 10 days (‘E11.5 + 10d’: Fig. 3A), contain THP‐positive loops of Henle (Fig. 3B). The very flat nature of these cultured kidneys made it possible to view the complete lengths of loops and collecting ducts in a way that was not revealed by cryosectioned kidneys isolated directly from embryos. The images demonstrated an apparent spatial association between the loops and the ducts, the loops running along the ducts, at least in the medulla (Fig. 3C). The images also allowed clear observation of the formation of distinct thick and thin segments of the loops (Fig. 3D). A time‐course study revealed that loops of Henle were only rarely detected at E11.5 + 6d but are common from E11.5 + 7d. By E11.5 + 10d, there was a mean of 16.2 ± 1.5 loops/kidney (‘±’ refers to standard deviation; five kidneys examined), and 78% had an error angle less than 45°, a figure broadly comparable to the 85% described for naturally developed kidneys (see above). The angles measured in these experiments are depicted graphically in Supporting Information Fig. S1a and further images are shown in Supporting Information Fig. S2.

### Loops of Henle orientation are adaptive and not set by nephron orientation

**Figure 4:**
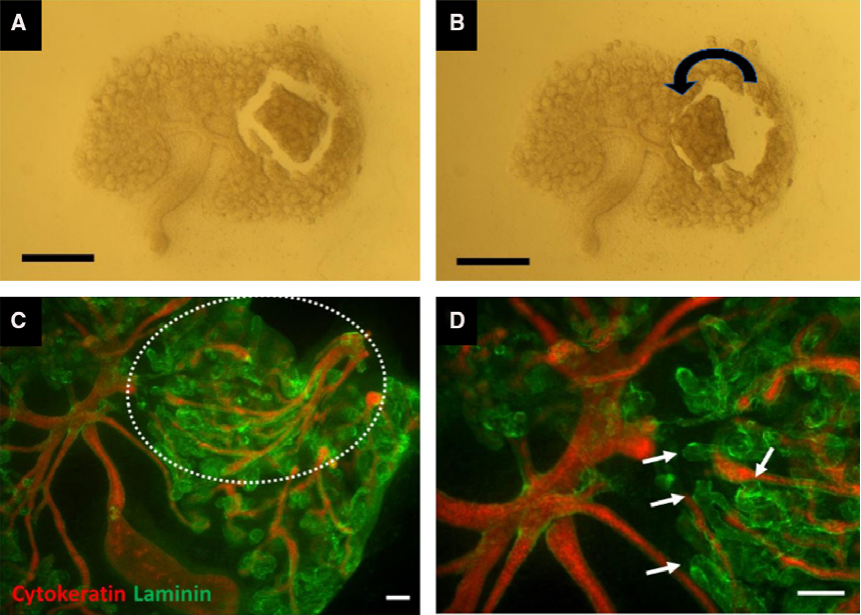
Loop of Henle orientation depends more on long‐range than on local cues. (A,B) Methods used. (A) At E11.5 +7d, a small piece of cortex is isolated by cutting round it, and then (B) rotated to reverse it along the cortico‐medullary axis. The gap where the cut was made appears spontaneously as the tissue springs back when cut, suggesting it was under tension during development. (C) Image of the same kidney after culture, showing the effect of this reversal: after 3 more days of culture, the collecting duct tree (red: pan‐cytokeratin) in the rotated portion of cortex (white dotted line) shows reverse orientation, branching towards the centre of the organ as a whole. A high‐power view of this rotated piece (D) shows loops of Henle (arrows) orientated completely wrongly with respect to their source tissue (reversed – growing towards and beyond the tips of the collecting ducts in the rotated piece), but correctly with respect to the organ as a whole. Scale bar: 500 μm (A,B), 40 μm (C,D). Additional images from this series of experiments are shown in Fig. S2.

The centripetal orientation of growing loops of Henle might arise from two types of influence, acting alone or in combination. One is the orientation of the S‐shaped body that gives rise to the loop, which is in turn set by the orientation of the ureteric bud tips in the cortex (reviewed by Saxén, 1987): a growing loop of Henle may be programmed to emerge from the S‐shaped body in a particular direction relative to that body and simply keep going. The other possible influence is a long‐range cue, for example, a chemoattractant released by the deep medulla of the kidney or a repellent released by the outer cortex. To discriminate between these possibilities, we cut a small piece of cortex out of our kidney cultures and replaced it, reversed along the cortico‐medullary axis (Fig. 4A,B). When the piece of cortex was reversed so that the ureteric tips within it now pointed towards the medulla of the kidney as a whole, and its developing nephrons were ‘upside‐down’ with respect to the organ as a whole, 94 ± 12% (of 18 loops, from four kidneys) of the loops of Henle ignored the reversed orientation of their own piece of tissue and migrated towards the medulla of the organ as a whole (Fig. 4C,D). The angles measured in these experiments are depicted graphically in Fig. S1b.

**Figure 5:**
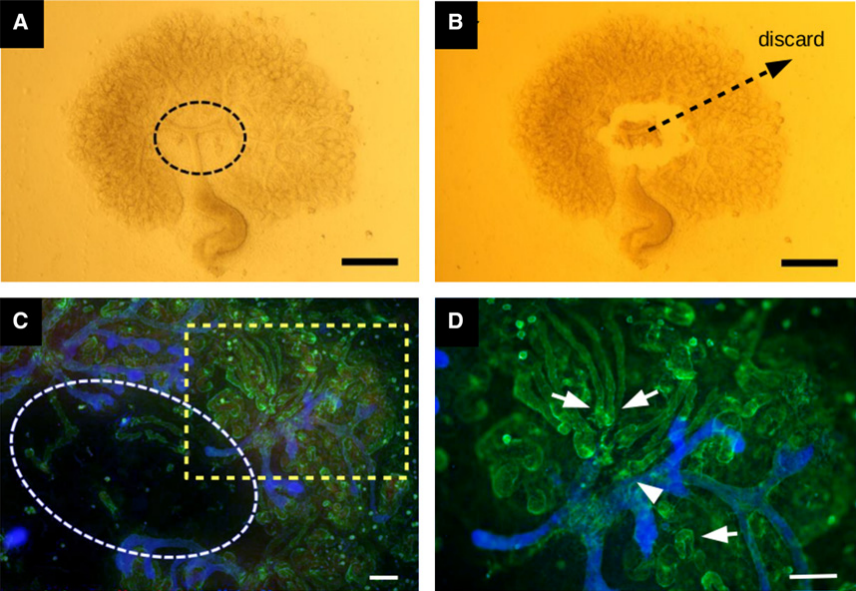
If the medulla is removed, loops of Henle orientate towards the largest remaining collecting ducts. (A,B) A typical medulla excision, performed at E11.5 + 7d. (C) A medulla‐less kidney incubated for a further 3 days, the ‘hole’ where the medulla was being indicated by a white dotted line, and the area shown at higher magnification in (D) is indicated by a yellow dotted line. (D) Convergence of loops of Henle on the largest (most mature) remaining collecting ducts (arrows). (C,D) The blue stain is anti‐calbindin D28k, a marker of collecting ducts, and the green anti‐laminin, a marker of all basement membranes of all epithelial tubules. Scale bars: 500 μm (A,B), 40 μm (C,D).

**Figure 6:**
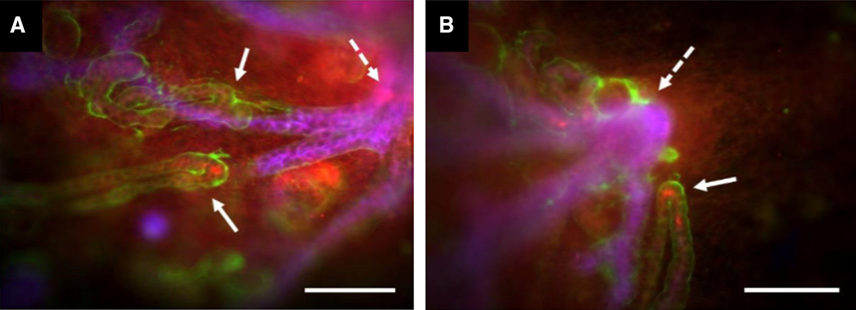
Loop of Henle orientation in isolated pieces of cortex. Images (A) and (B) are two examples of small pieces of cortex isolated from cultured kidneys at E11.5 + 7d and then cultured for 3 more days. The orientation of the piece of tissue (in both images, the old edge of the kidney being to the left) is indicated by the branching collecting duct system (blue – CalbindinD^28k^). Examples of loop of THP‐positive (red) Henle tips are highlighted in solid arrows, and the oldest/largest remaining branch points of the collecting duct system are indicated with dotted arrows. The loops appear to be heading towards these oldest/largest branch points. Scale bar: 200 μm.

The above finding strongly suggests the presence of long‐range signalling. It does not prove there is no local signalling but does show that, even if local influences are there, long‐range signals over‐ride them. We attempted to identify the anatomical source of the long‐range signals by removing pieces of the medulla (Fig. 5A,B). It is interesting to note in passing that the medulla, when cut, always (in four of four kidneys) sprang back to a smaller shape, suggesting it was under tension before cutting. When the medulla was removed, loops of Henle did not grow into the vacant space but instead converged on the nearest remaining large branch point of the collecting duct system (Fig. 5C,D; quantitative information on these experiments is shown in Supporting Information Fig. S3a). This effect was seen however large a portion of the medulla was removed. Indeed, it was seen when a piece of cortex was isolated and cultured on its own: the loops of Henle converged on the largest collecting duct branch point available within that piece of cortex (Fig. 6A,B; three of three kidneys; quantitative information on these experiments is shown in Fig. S3b).

## Discussion

The data in the Results section show that, from their earliest emergence, loops of Henle grow in a direction orientated towards the centre of the kidney, and their accuracy improves with maturity. This orientation is also seen in organ culture, and manipulations of the cultures indicate that the loops seem to respond to long‐range cues rather than to the orientation of their parent nephrons or to cues local to the cortex. The loops normally grow toward the centre of the kidney, where the largest and most mature collecting duct branch (future renal pelvis) lies: if deprived of that target, they orientate towards the largest (most mature) collecting duct branch point available to them.

The simplest explanation for these findings is that, as the collecting duct matures, it, or possibly stromal cells intimately associated with it, release diffusible signalling molecules that regulate the growth of the loop of Henle tubules. Young collecting ducts, in the cortex, do not seem to do this: if they did, their proximity to developing loops of Henle would make them a much greater influence than the more distant medulla. Given that, in the absence of deep medulla, the loops navigated to the largest (oldest) available duct branch point, there may be a gradient of factor production, strongest in the deep medulla, weaker but detectable in the outer medulla/ inner cortex, and insignificant in the outer cortex.

How might a diffusible signalling molecule guide the extension of a looped tube? Analogies with other systems suggest two broad types of mechanism, which might operate independently or together. One possible mechanism, the ‘pull’ mechanism, is analogous to navigation by *Drosophila* tracheae or mammalian capillary sprouts. In it, navigation would be performed by one or more specialized ‘tip cells’, in this case at the apex of the loop. In insect tracheal and mammalian capillary growth, tip cells extend lamellipodia and filopodia to sample the environment ahead of them: the lamellipodial/filopodial extensions encountering the highest level of chemoattractant are the most stable and exert the most traction, dragging the cell and therefore the tube behind it in that direction (Caussinus et al. 2008, for tracheae; Gerhardt et al. 2004, for capillaries). This orientates the whole tube in that direction. In the tracheal system, elongation is achieved by convergent extension of stalk cells behind the tip, in response to the pulling of the tip (Caussinus et al. 2008). The main objection to this being the mechanism for loop of Henle orientation is that there is no evidence for any special ‘tip’ cells existing. In our images, and the images of others (Cha et al. 2001; Pietilä et al. 2011, which are among the few publications that contain images in which the apex of the loop can be discerned), there is no consistent evidence for the apex of the loop extending any exploratory structures; at most, one can see wisps of laminin associated with the apex of an occasional rare loop (they are visible at the ends of the right‐most loop of Fig. 3B and the right‐most loop of Fig. 3C in this report).

The second possible mechanism, the ‘push’ mechanism, would operate by modulation of growth processes (extent or orientation of cell division) along the shafts of the loop and may be seen as broadly analogous to phototropism in plants (reviewed by Liscum et al. 2014). There is little direct information on how loops of Henle elongate, although it is known that proliferation takes place during elongation and is more frequent in the outer medullary and inner cortical portions of the loop and less in the inner medullary portion (rat data: Cha et al. 2001). Growth of mouse renal collecting ducts, which run approximately parallel to the loops of Henle, have been studied more closely than the loops: these ducts grow by a combination of cell proliferation (not orientated until post‐natal growth: Karner et al. 2009) and cell intercalation/convergent extension (active from early stages: Karner et al. 2009). Assuming either or both of these operates in the loop, they would create a ‘push’ to drive the apex onward from behind. If a loop were heading precisely in the direction of the medulla – the source of a growth‐inhibiting signal – both sides of it would elongate at the same rate and it would grow straight, its growth slowing as it encountered higher and higher concentrations of the signal until growth stopped some way short of the first branch point of the collecting duct (future renal pelvis). The reduced frequency of cell divisions in the deep medullary part of the loop compared with outer parts, reported by Cha et al. (2001), supports the idea of the medulla making a proliferation‐inhibiting signal. If the loop were heading at an angle to its proper path, the side closest to the medulla would receive a higher concentration of the signal and so would grow more slowly than the opposite side: the differential growth would result in the tube bending until it was again in the correct direction. This type of mechanism seems promising in that it does not invoke any specialized cell types for which there is no evidence, and it could explain both navigation by the loops and the eventual cessation of their growth. The main objection to it is that it would leave traces of error correction as bends in the loops, but loops of Henle are typically depicted as running straight when drawn in textbooks. It is noticeable, however, that many loops seen in our organ culture experiments, which allow observation of the whole loop rather than merely sections of it, do show gradual bending, just like stems of phototropic plants (see, for example, our Fig. 5D).

We have not identified the signalling molecule(s) involved in guiding the loop of Henle, and doing so would be beyond the scope of this anatomical/embryological report. The reorientation of loops towards the whole kidney medulla when a piece of cortex is rotated does, however, strongly support the idea of a chemical rather than a mechanical signal (as mechanical connections were severed in the experiment). The expected phenotype of a loss‐of‐function mutant in a loop‐guiding signalling pathway would be randomization of loops of Henle. To the best of our knowledge, no such phenotype has been described. The mutation with the most dramatic effect on medulla formation, a kidney‐specific knockout of Wnt7b (Yu et al. 2009), affects the extent of loop elongation but the short loops still do not orientate incorrectly with any, for example, heading out outwards the renal periphery (this correct orientation can be seen in Fig. 5B of the Yu et al. paper). It may of course be that the lost‐loop phenotype cannot be observed because the signalling pathway in question is required earlier in kidney or embryonic development, so renal development fails before loop navigation begins. One way of proceeding to identify the signal(s) might be to examine the GUDMAP database of gene expression in developing kidneys (Little et al. 2007; Thiagarajan et al. 2011a,b; Davies et al. 2012) for ligand‐receptor pairs, in which the ligand is expressed in medullary (and not outer‐cortical) collecting duct, and the receptor by the loop of Henle, and then to use drugs, function‐blocking antibodies and ectopic sources of the ligand to try to perturb natural navigation. This approach has been proved successful in identifying a molecule involved in navigation by collecting duct tips (Davies et al. 2014), and we intend to apply it to the loop of Henle problem in the near future.

## Supporting information


**Fig. S1.** Quantitative analysis of loop directions in the experiments depicted in main text Figs 3 and 4.
**Fig. S2.** Additional pictures of loop reorientation, as shown in Fig. 4 in the main text.
**Fig. S3.** Quantitative analysis of loop directions in the experiments depicted in main text Figs 5 and 6.
