# Supplementary material for: In developing mouse kidneys, orientation of loop of Henle growth is adaptive and guided by long‐range cues from medullary collecting ducts
Source: J Anat. 2019 May 17;235(2):262–70. doi: 10.1111/joa.13012 (PMC6637448; doi:10.1111/joa.13012)
Supplement: Supplementary file 1 — Fig. S1. Quantitative analysis of loop directions in the experiments depicted in main text Figs 3 and 4. Fig. S2. Additional pictures of loop reorientation, as shown in Fig. 4 in the main text. Fig. S3. Quantitative analysis of loop directions in the experiments depicted in main text Figs 5 and 6. [file JOA-235-262-s001.docx]

**SUPPLEMENTARY MATERIAL**

**
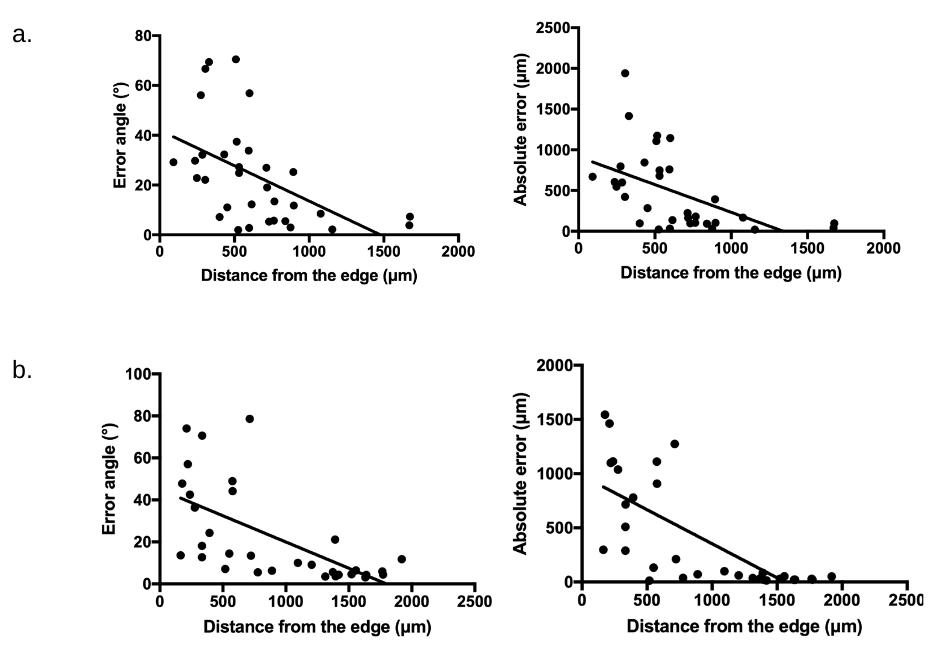
**

**Figure S1:** Quantitative analysis of loop directions in the experiments depicted in main text figures 3 and 4. The graphs in (a) show the accuracy of loop navigation in organ culture, in terms of error angle and absolute error (these measures are explained in the main text). Accuracy in terms of error angle, and the tendency to higher accuracy with greater distance from the edge, is similar to that in vivo (main Fig 2e), though absolute error is larger, perhaps reflecting the wider, flatter form of kidneys in culture. The graphs in (b) show the accuracy of loop navigation towards the central collecting duct tissues of a kidney, by loops of Henle emerging from rotated cortex as in main Fig 4. The accuracy is broadly similar to that seen in normal cultured kidneys (a, this figure).

**
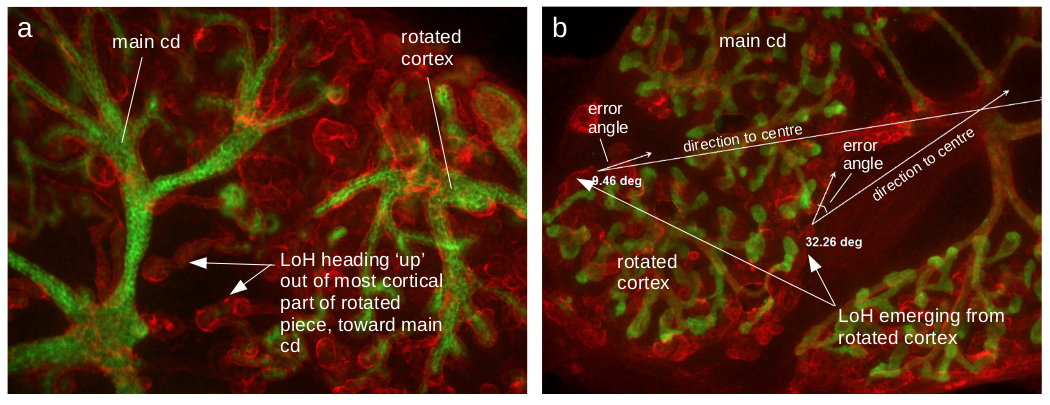
**

**Figure S2**: Additional pictures of loop reorientation, as shown in Figure 4 in the main text. (a) shows an example of loops of Henle (‘LoH’) extending long distances ‘the wrong way’ from their local rotated cortex (out past the local collecting duct tips) towards the older CD branches of the main collecting duct (‘cd’) tree. (b) shows a similar experiment at lower magnification, with construction lines for measuring error angles superimposed on the image.

**
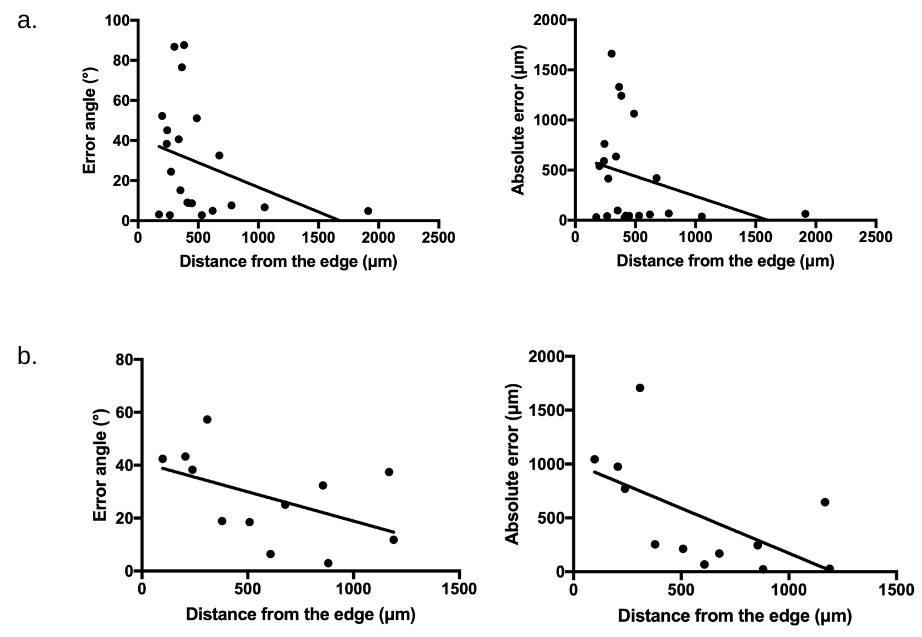
**

**Figure S3:** Quantitative analysis of loop directions in the experiments depicted in main text figures 5 and 6. The graphs in (a) show the accuracy of loop navigation towards the nearest remaining large collecting duct, when the medulla of a kidney has been removed as depicted in main figure 5. The graphs in (b) show the accuracy of loop navigation towards largest remaining large collecting duct in a piece of isolated cortex. The accuracy is similar to normal cultured kidneys, though the spread of data points is greater (possibly reflecting inevitable variation between dissections, all of which were performed manually). In both of these panels, the ‘edge’ used for distance is the outer edge of the cortex; new edges created by the dissection are ignored (as they would not be a proxy of nephron age, as distance from the real outer edge of the cortex is).
